# Survival Outcomes Among Patients With Hepatocellular Carcinoma in a Large Integrated US Health System

**DOI:** 10.1001/jamanetworkopen.2024.35066

**Published:** 2024-09-24

**Authors:** Mignote Yilma, Richie Houhong Xu, Varun Saxena, Monica Muzzin, Lue-Yen Tucker, Jeffrey Lee, Neil Mehta, Nizar Mukhtar

**Affiliations:** 1 General Surgery, University of California, San Francisco; 2 National Clinician Scholars Program, San Francisco, California; 3 Division of Research, Kaiser Permanente, Oakland, California; 4 Department of Gastroenterology, Kaiser Permanente South San Francisco Medical Center, San Francisco, California; 5 Department of Medicine, University of California, San Francisco; 6 Department of Gastroenterology, Kaiser Permanente San Francisco Medical Center, San Francisco

## Abstract

**Importance:**

Hepatocellular carcinoma (HCC) is the leading oncologic cause of death among patients with cirrhosis, but large studies examining mortality trends are lacking.

**Objective:**

To evaluate survival among patients with HCC in one of the largest integrated health care systems in the US.

**Design, Setting, and Participants:**

This retrospective cohort study included 3441 adult patients who received a diagnosis of HCC between January 1, 2006, and December 31, 2019, with end of follow-up on December 31, 2020. The study period was further categorized as era 1, defined as 2006 to 2012, and era 2, defined as 2013 to 2019. Statistical analysis was conducted from January 2021 to June 2024.

**Exposures:**

Patient demographic characteristics and disease factors.

**Main Outcomes and Measures:**

All-cause and HCC-specific mortality were used as primary end points, and survival probabilities were estimated using the Kaplan-Meier method. Cox proportional hazards regression analyses were adjusted for age at diagnosis, sex, race and ethnicity, cause of disease, Barcelona Clinic Liver Cancer (BCLC) stage, alpha-fetoprotein level, and treatment type.

**Results:**

Of 3441 patients with HCC, 2581 (75.0%) were men, and the median age was 65 years (IQR, 58-73 years). A total of 1195 patients (34.7%) received curative treatment, 1374 (39.9%) received noncurative treatment, and 872 (25.3%) received no treatment. During the study period, 2500 patients (72.7%) experienced all-cause mortality, and 1809 (52.6%) had HCC-specific mortality. In multivariable analysis, being 70 years of age or older (adjusted hazard ratio [AHR], 1.39; 95% CI, 1.22-1.59), male sex (AHR, 1.20; 95% CI, 1.07-1.35), BCLC stage C or D (AHR, 2.40; 95% CI, 2.15-2.67), increasing alpha-fetoprotein level (vs <20 ng/mL; 20-99 ng/mL: AHR, 1.20; 95% CI, 1.04-1.38; ≥1000 ng/mL: AHR, 2.84; 95% CI, 2.45-3.25), noncurative treatment (AHR, 2.51; 95% CI, 2.16-2.90), and no treatment (AHR, 3.15; 95% CI, 2.64-3.76) were associated with higher all-cause mortality, while Asian or Other Pacific Islander race and ethnicity (vs non-Hispanic White; AHR, 0.76; 95% CI, 0.65-0.88) was associated with lower all-cause mortality. Survival improved in diagnosis era 2 (2013-2019; n = 2007) compared with diagnosis era 1 (2006-2012; n = 1434).

**Conclusions and Relevance:**

This large, racially and ethnically diverse cohort study of patients with HCC found improving survival over time, especially among individuals with early-stage HCC receiving potentially curative treatments. This study highlights the importance of surveillance for detection of HCC at early stages, particularly among groups at risk for poorer outcomes.

## Key Points

**Question:**

How has hepatocellular carcinoma survival changed over time, and what factors shape clinical outcomes?

**Findings:**

This cohort study of 3441 adults with hepatocellular carcinoma showed that while overall survival remains low, there have been significant improvements in disease-specific and overall survival over the past decade, particularly among patients with early-stage disease undergoing curative treatments.

**Meaning:**

These findings suggest that detection of hepatocellular carcinoma at early stages is critical to ensuring patient survival; additional demographic and clinical features can be used to identify patients at high risk for poor outcomes and inform targeted interventions.

## Teaser

This cohort study evaluates survival and the factors that shape clinical outcomes among patients with hepatocellular carcinoma in one of the largest integrated health care systems in the US.

## Introduction

Hepatocellular carcinoma (HCC) is the most common cause of cancer-related morbidity and mortality globally,^1,2^ and it is the leading oncologic cause of death among patients with cirrhosis. The incidence of HCC has more than doubled in the US over the past 2 decades, and this trend is expected to continue over the next 20 years.^3,4,5^ Although an increasing number of treatment options and improved patient selection^6,7^ have led to modest improvements in outcomes, HCC remains a difficult-to-treat cancer.^8^

The 2022 Barcelona Clinic Liver Cancer (BCLC) staging and treatment strategy recognizes the heterogenous nature of HCC and the evolving landscape of treatment options, particularly for intermediate-stage disease (BCLC stage B).^8^ These patients are now candidates for several treatment options,^8^ including curative therapies, such as liver transplant (LT)^9^ and noncurative locoregional and systemic therapies, and multiple treatment modalities are often used in their care.^8^ Downstaging HCC to within Milan criteria for LT has emerged as a reliable approach with comparable post-LT survival estimates.^8^ In recent years, the increasing use of locoregional therapy, such as yttrium-90 (Y90) radioembolization,^10,11^ and newer systemic immunotherapy treatment options has significantly improved survival estimates for patients with BCLC stages B and C.^12,13,14,15,16,17,18,19^ However, large clinical studies evaluating treatment outcomes across all HCC stages in this dynamic era are lacking.

Available data regarding HCC management outcomes stem largely from small, single academic medical center experiences with limited generalizability to the community practice setting or large national database analyses that lack granularity.^20,21,22^ To address this gap, we evaluated factors associated with all-cause mortality and survival trends among one of the largest HCC cohorts described to date receiving care in a large integrated health care system in the US.

## Methods

### Study Design

We conducted a retrospective cohort study in full compliance with the Declaration of Helsinki.^23^ The study was approved by the institutional review board at Kaiser Foundation Research Institute with a waiver of consent because patient data were deidentified. The study followed the Strengthening the Reporting of Observational Studies in Epidemiology (STROBE) reporting guideline.^24^

### Data Collection

Patients who initially received a diagnosis of HCC between January 1, 2006, and December 31, 2019, were identified through the Kaiser Permanente Northern California (KPNC) Cancer Registry using the *International Classification of Diseases for Oncology, Third Edition* diagnosis codes for HCC (81703, 81723, 81733, 81743, and 81753) and were followed up through December 31, 2020 (administrative study end date). The study period was divided into 2 eras based on the year of HCC diagnosis: era 1 comprised years 2006 to 2012, and era 2 comprised years 2013 to 2019. This categorization was selected for several reasons: (1) it bifurcates the study period evenly, (2) the years 2011 to 2013 marked the availability of direct-acting antivirals for treatment of chronic hepatitis C,^25^ and (3) the years 2011 to 2013 saw the first major update to the Liver Imaging Reporting and Data System to align with the Organ Procurement and Transplantation Network policy.^26^ Baseline demographic and clinical characteristics were indexed on the initial HCC diagnosis date. Patients aged 17 years or younger at the time of HCC diagnosis and those who received a diagnosis of other malignant cancers within 5 years were excluded from the study.

TNM as recorded in the KPNC Cancer Registry, Child-Turcotte-Pugh class, and other clinical elements were used to derive BCLC stage. Child-Turcotte-Pugh class was determined using laboratory values within 1 year of HCC diagnosis (but before HCC treatment), prior diagnosis of hepatic encephalopathy (based on *International Classification of Diseases, Ninth Revision, Clinical Modification* [*ICD-9-CM*] and *International Statistical Classification of Diseases, Tenth Revision, Clinical Modification* [*ICD-10-CM*] codes or prescription for lactulose), and prior diagnosis of ascites based on *ICD-9-CM* or *ICD-10-CM* codes or prescription for spironolactone. Treatment modalities were extracted from electronic health records (EHRs) using *Current Procedural Terminology* codes for transarterial chemoembolization, transarterial radioembolization (TARE), percutaneous ethanol injection, Y90 radioembolization, cyberknife, stereotactic radiosurgery or stereotactic body radiotherapy, thermal ablation, and surgical resection. Liver transplant data were obtained from the Phoenix database, and receipt of systemic therapy was based on having at least 1 filled prescription for sorafenib, lenvatinib, nivolumab, pembrolizumab, regorafenib, cabozantinib, or ramucirumab. All medication prescriptions were determined via outpatient pharmacy records by using pharmacy dispensary dates. Structured EHR review was performed to validate electronically extracted treatment data. Treatment dates and modalities included those from the date of diagnosis to study end points.

Data pertaining to year of diagnosis, demographic characteristics (age at diagnosis, sex, and race and ethnicity), BCLC stage, alpha-fetoprotein (AFP) level, treatment type, and survival follow-up (cause of death and survival months) were included for analysis. Patient demographic characteristics, cause of disease, and BCLC stage were collected at the time of HCC diagnosis. Alpha-fetoprotein measurements within 1 year of HCC diagnosis and prior to any HCC treatment were collected.

### Study Variables

Race and ethnicity data were assessed to identify HCC-related health disparities. Self-identified race and ethnicity categories, based on EHR data, included Hispanic or Latinx, non-Hispanic or Latinx Asian or Other Pacific Islander (hereafter, *Asian or Other Pacific Islander*), non-Hispanic or Latinx Black (hereafter, *Black*), non-Hispanic or Latinx White (hereafter, *White*), and other race and ethnicity (American Indian or Alaska Native, multiracial patients, or patients of unknown race and ethnicity). The cause of disease was identified based on *ICD-9-CM* or *ICD-10-CM* codes, including chronic hepatitis C (HCV), chronic hepatitis B (HBV), alcohol-related liver disease, nonalcoholic fatty liver disease, and unknown or other cause of disease. Structured EHR review of the patients’ clinical histories were conducted by one of us (M.Y.) to determine the cause of the disease for patients whose cause could not be determined based on *ICD-9-CM* or *ICD-10-CM* codes. The “other” cause of disease category comprises primary biliary cholangitis, primary sclerosing cholangitis, hereditary hemochromatosis, autoimmune hepatitis, cryptogenic, Budd-Chiari syndrome, polycystic liver disease, and unknown cause. Barcelona Clinic Liver Cancer stage was categorized into 0 and A (very early or early stage), B (intermediate stage), C (advanced stage), and D (terminal stage) for survival estimates and was categorized into advanced (BCLC stages C and D) and nonadvanced (BCLC stage 0, A, and B) for Cox proportional hazards regression analysis. Alpha-fetoprotein level was categorized into lower than 20 ng/mL, 20 to 99 ng/mL, 100 to 999 ng/mL, and 1000 ng/mL or higher (to convert to micrograms per liter, multiply by 1.0) based on previous studies on HCC prognosis and recurrence.^27,28^ Treatment was categorized as no treatment, noncurative treatment, and curative treatment. Noncurative treatment included transarterial chemoembolization, TARE, Y90 radioembolization, cyberknife, stereotactic body radiotherapy, and systemic chemotherapy. Curative treatment included LT, surgical resection, and thermal ablation.

### Outcomes

The major outcomes explored in this study were (1) survival probability stratified by study era, BCLC stage at time of diagnosis, and treatment group and (2) 5-year hazard ratios (HRs) for all-cause and HCC-specific survival, adjusted for age at diagnosis, sex, race and ethnicity, cause of disease, BCLC stage, AFP level, and treatment type. Hepatocellular carcinoma–specific cause of death was obtained from the National Death Index or the state of California vital records.

### Censoring

Mortality data were gathered from the state of California vital records, National Death Index, and the KPNC EHR. All-cause mortality was defined as the time interval between initial HCC diagnosis and death due to any cause. Hepatocellular carcinoma–specific mortality was defined as the interval between initial HCC diagnosis and death due to HCC-related causes (identified by *ICD-10-CM* codes C22.0, C22.9, C78.7, C37.6, and D37.6). At the time of data collection and analysis, all-cause mortality data were complete through 2020, and HCC-specific mortality data were complete through 2019. Patients who did not experience death by the date they lost KPNC membership or the administrative study end date were censored.

### Statistical Analysis

Statistical analysis was conducted from January 2021 to June 2024. Patient characteristics were summarized as frequencies and proportions for categorical variables. Median values and IQRs were used to report continuous data.

Survival probabilities were calculated using the Kaplan-Meier method and compared using log-rank tests. Our primary survival analysis was by diagnosis era (era 1: 2006-2012; era 2: 2013-2019), and our secondary survival analyses were by BCLC stage and treatment group. We performed exploratory subgroup ad hoc analyses of study era by BCLC stage and treatment group.

Univariable and multivariable Cox proportional hazards regression analyses were used to identify factors associated with all-cause and HCC-specific mortality at 1 and 5 years of follow-up. Patients were followed up from the initial HCC diagnosis date until the earliest date of death, end of KPNC membership, or administrative study end date. For HCC-specific mortality, competing-risk Cox proportional hazards regression models were developed to account for non–HCC-specific mortality. Treatment groups were modeled as time-dependent covariates updated over time as patients received new treatment.

All statistical tests for differences were performed with the threshold of significance set at a 2-sided *P* < .05. Statistical analyses and data visualizations were performed using SAS, version 9.4 (SAS Institute Inc).

## Results

### Patient Characteristics

Of 3441 adult patients who received a diagnosis of HCC between 2006 and 2019, 2581 (75.0%) were male and 860 (25.0%) were female, and the median age was 65 years (IQR, 58-73 years) (Table 1). Race and ethnicity of the cohort comprised 847 Asian or Other Pacific Islander patients (24.6% ), 288 Black patients (8.4%), 754 Hispanic or Latinx patients (21.9%), 1465 White patients (42.6%), and 87 patients (2.5%) of other race and ethnicity. Chronic HCV (1543 [44.8%]) and HBV (514 [14.9%]) infection were the predominant causes of liver disease, and 2582 of the study cohort (75.0%) had cirrhosis. Most patients had BCLC stage 0 or A (2062 [59.9%]), with a higher proportion in era 2 compared with era 1 (1274 of 2003 [63.6%] vs 788 of 1421 [55.5%]; *P* < .001). More than one-fourth of the cohort (965 [28.0%]) had advanced-stage disease (BCLC stage C or D) at diagnosis, with a slightly lower proportion in era 2 compared with era 1 (548 of 2007 [27.3%] vs 417 of 1434 [29.1%]; *P* < .001). Serum AFP levels were elevated at 20 ng/mL or higher among more than half (1734 [50.4%]) of the cohort, with a lower proportion in era 2 compared with era 1 (943 of 2007 [47.0%] vs 791 of 1434 [55.2%]; *P* < .001). Three-quarters of patients in this cohort (2569 [74.7%]) received at least 1 treatment for HCC after diagnosis; 1374 (39.9%) received noncurative treatment only, 1195 (34.7%) received curative treatment, and 872 (25.3%) received no treatment. During the study period, 2500 patients (72.7%) experienced all-cause mortality, and 1809 (52.6%) had HCC-specific mortality.

**Table 1.** Characteristics of Patients With HCC at Kaiser Permanente Northern California, 2006-2019

Variable	Overall (N = 3441), No. (%)^a^	Era 1 (2006-2012) (n = 1434), No. (%)	Era 2 (2013-2019) (n = 2007), No. (%)	*P* value
Categorical age at HCC diagnosis, y				
18-39	39 (1.1)	19 (1.3)	20 (1.0)	<.001
40-59	993 (28.9)	546 (38.1)	447 (22.3)	
60-69	1275 (37.1)	421 (29.4)	854 (42.6)	
≥70	1134 (33.0)	448 (31.2)	686 (34.2)	
Age, median (IQR)	65 (58-73)	63 (56-72)	66 (60-73)	<.001
Sex				
Male	2581 (75.0)	1080 (75.3)	1501 (74.8)	.72
Female	860 (25.0)	354 (24.7)	506 (25.2)	
Race and ethnicity				
Asian or Other Pacific Islander	847 (24.6)	360 (25.1)	487 (24.3)	.02
Black	288 (8.4)	139 (9.7)	149 (7.4)	
Hispanic or Latinx	754 (21.9)	287 (20.0)	467 (23.3)	
White	1465 (42.6)	605 (42.2)	860 (42.9)	
Other^b^	87 (2.5)	43 (3.0)	44 (2.2)	
Diabetes (type 1 or 2)	1439 (41.8)	578 (40.3)	861 (42.9)	.13
Chronic kidney disease stage				
Stage 1 (normal)	1450 (42.1)	606 (42.3)	844 (42.1)	.78
Stage 2 (mild)	1292 (37.5)	539 (37.6)	753 (37.5)	
Stage 3 (moderate)	559 (16.2)	225 (15.7)	334 (16.6)	
Stage 4 (severe)	91 (2.6)	40 (2.8)	51 (2.5)	
Stage 5 (failure)	49 (1.4)	24 (1.7)	25 (1.2)	
Cause of liver disease				
Chronic HCV	1543 (44.8)	642 (44.8)	901 (44.9)	<.001
Chronic HBV	514 (14.9)	224 (15.6)	290 (14.4)	
Nonalcoholic fatty liver disease	351 (10.2)	96 (6.7)	255 (12.7)	
Alcohol-related liver disease	324 (9.4)	140 (9.8)	184 (9.2)	
Other or unknown^c^	709 (20.6)	332 (23.2)	377 (18.8)	
Diagnosis of cirrhosis or fibrosis-4 index >3.25	2582 (75.0)	1048 (73.1)	1534 (76.4)	.03
BCLC stage				
Very early or early stage (0 or A)	2062 (59.9)	788 (55.0)	1274 (63.5)	<.001
Intermediate stage (B)	396 (11.5)	215 (15.0)	181 (9.0)	
Advanced stage (C)	714 (20.8)	316 (22.0)	398 (19.8)	
Terminal stage (D)	252 (7.3)	102 (7.1)	150 (7.5)	
Advanced BCLC stage (C or D)	965 (28.0)	417 (29.1)	548 (27.3)	.01
Categorical alpha-fetoprotein level, ng/mL				
<20	1543 (44.8)	532 (37.1)	1011 (50.4)	<.001
20-99	600 (17.4)	255 (17.8)	345 (17.2)	
100-999	557 (16.2)	263 (18.3)	294 (14.6)	
≥1000	577 (16.8)	273 (19.0)	304 (15.1)	
Treatment type				
No treatment	872 (25.3)	465 (32.4)	407 (20.3)	<.001
Noncurative only	1374 (39.9)	564 (39.3)	810 (40.4)	
Curative^d^	1195 (34.7)	405 (28.2)	790 (39.4)	
Treatment type (most to least aggressive)				
Liver transplant (most aggressive)	338 (9.8)	157 (10.9)	231 (11.5)	<.001
Resection	357 (10.4)	113 (7.9)	244 (12.2)	
Thermal ablation	450 (13.1)	135 (9.4)	315 (15.7)	
Yttrium-90 radioembolization	125 (3.6)	2 (0.1)	123 (6.1)	
TACE or TARE	939 (27.3)	393 (27.4)	546 (27.2)	
Cyberknife or SBRT	28 (0.8)	11 (0.8)	17 (0.8)	
Systemic chemotherapy	282 (8.2)	158 (11.0)	124 (6.2)	
No treatment	872 (25.3)	465 (32.4)	407 (20.3)	
All-cause mortality	2500 (72.7)	1253 (87.4)	1247 (62.1)	<.001
HCC-related mortality	1809 (52.6)	953 (66.5)	856 (42.7)	<.001

Abbreviations: BCLC, Barcelona Clinic Liver Cancer; HBV, hepatitis B virus; HCC, hepatocellular carcinoma; HCV, hepatitis C virus; SBRT, stereotactic body radiotherapy; TACE, transarterial chemoembolization; TARE, transarterial radioembolization.

SI conversion factor: To convert alpha-fetoprotein to micrograms per liter, multiply by 1.0.

^a^ Missing data: alpha-fetoprotein, 164; and BCLC stage, 17.

^b^ Includes American Indian or Alaska Native, multiracial, and unknown.

^c^ Includes metabolic or autoimmune (n = 53), cryptogenic (n = 12), Budd-Chiari syndrome (n = 1), polycystic liver disease (n = 1), and other unknown cause (n = 642).

^d^ Curative treatment indicates ablation, resection, and transplant.

In comparing trends in treatment over time, a higher proportion of patients received curative treatment in era 2 compared with era 1 (790 of 2007 [39.4%] vs 405 of 1434 [28.2%]; *P* < .001), including LT (231 of 2007 [11.5%] vs 157 of 1434 [10.9%]), surgical resection (244 of 2007 [12.2%] vs 113 of 1434 [7.9%]), and thermal ablation (315 of 2007 [15.7%] vs 135 of 1434 [9.4%]) (Table 1). A lower proportion of patients received systemic therapy (124 of 2007 [6.2%] vs 158 of 1434 [11.0%]) and no HCC-directed treatment (407 of 2007 [20.3%] vs 465 of 1434 [32.4%]) in era 2 compared with era 1 (*P* < .001). For patients with at least 5 years of follow-up time from their initial HCC diagnosis, the median follow-up time until death or loss of coverage was 1.3 years (IQR, 0.3-5.0 years).

### Overall Survival by Study Era, BCLC Stage, and Treatment Type

**Figure 1.**
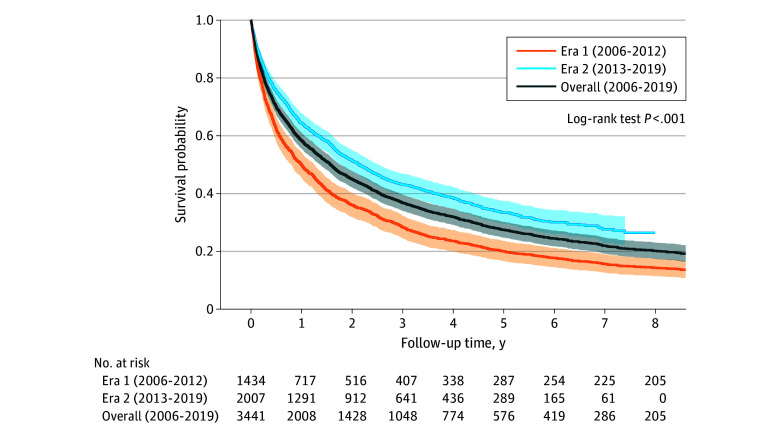
Kaplan-Meier Survival Curves of Hepatocellular Carcinoma Cohort Stratified by Diagnosis Era Plots are truncated after 8 years of follow-up. Median survival and 95% CIs are calculated based on all patients who are alive with minimal follow-up of 1 year and maximal follow-up of 15 years. Shaded areas indicate the 95% CIs.

**Figure 2.**
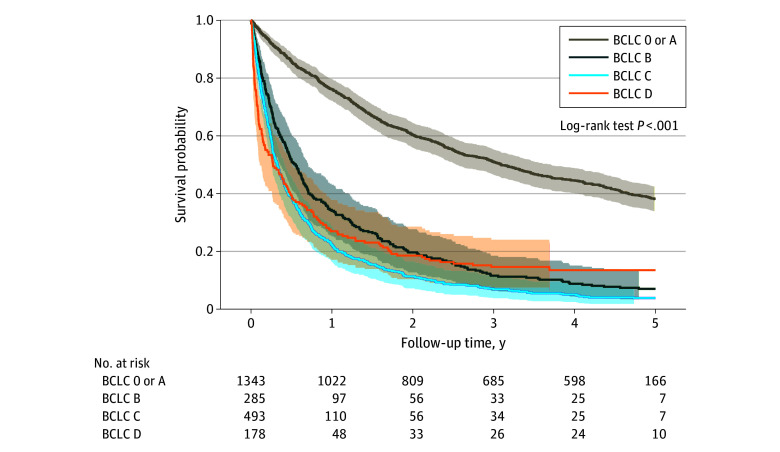
Kaplan-Meier Survival Curves of Hepatocellular Carcinoma Cohort Stratified by Barcelona Clinic Liver Cancer (BCLC) Stage Shaded areas indicate the 95% CIs.

Overall survival estimates improved between era 1 and era 2 (Figure 1). Survival estimates varied by BCLC stage at the time of diagnosis; patients with very-early or early-stage disease (BCLC 0 or A) had substantially higher survival probability compared with patients with intermediate-stage disease (BCLC B) or advanced-stage disease (BCLC C and D) at 5 years of follow-up (Figure 2). When comparing era 1 and era 2, improvements in survival estimates were observed across most stages of disease (eFigure 1 in Supplement 1).

**Figure 3.**
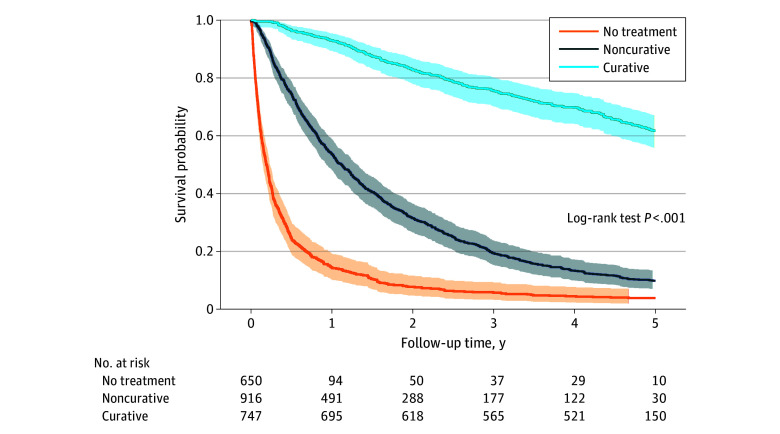
Kaplan-Meier Survival Curves by Treatment Group Plots are truncated after 8 years of follow-up. Median survival and 95% CIs are calculated based on all patients who are alive with minimal follow-up of 1 year and maximal follow-up of 15 years. Shaded areas indicate the 95% CIs.

Survival estimates differed significantly based on HCC treatment, such that patients who received no treatment had the worst survival probability compared with patients who received noncurative and curative treatments at 5 years (Figure 3). Comparing era 2 with era 1 and stratifying by treatment group, we observed higher 5-year survival probability among patients who received curative and noncurative treatment over time, while survival among patients who received no treatment did not vary significantly (eFigure 2 in Supplement 1).

### Factors Associated With HCC Survival

Univariate Cox proportional hazards regression analysis of factors associated with overall and HCC-specific survival are included in eTable 1 and eTable 2 in Supplement 1. Multivariable Cox proportional hazards regression models for all-cause mortality at 5 years of follow-up (Table 2) showed that being 70 years of age or older (adjusted HR [AHR], 1.39; 95% CI, 1.22-1.59), male sex (AHR, 1.20; 95% CI, 1.07-1.35), alcohol-related liver disease (vs chronic HCV; AHR, 1.34; 95% CI, 1.13-1.59), BCLC stage C or D (AHR, 2.40; 95% CI, 2.15-2.67), higher AFP level (vs <20 ng/mL; 20-99 ng/mL: AHR, 1.20; 95% CI, 1.04-1.38; ≥1000 ng/mL: AHR, 2.84; 95% CI, 2.48-3.25), noncurative treatment (vs curative treatment; AHR, 2.51; 95% CI, 2.16-2.90), and no treatment (vs curative treatment; AHR, 3.15; 95% CI, 2.64-3.76) were associated with higher overall mortality. Asian or Other Pacific Islander race and ethnicity (AHR, 0.76; 95% CI, 0.65-0.88) was associated with lower all-cause mortality relative to White race and ethnicity. Multivariable Cox proportional hazards regression models (eTable 3 in Supplement 1) for HCC-specific mortality among patients with 5 years of follow-up showed similar findings.

**Table 2.** Multivariable Cox Proportional Hazards Regression of All-Cause Mortality With 1 and 5 Years of Follow-Up From HCC Diagnosis

Variable	Adjusted HR (95% CI)	
	1-y Follow-up^a^	5-y Follow-up^b^
Age at HCC diagnosis, y (reference: 40 to <60)		
18 to <40	0.95 (0.53-1.71)	0.79 (0.47-1.34)
60 to <70	0.97 (0.84-1.12)	0.97 (0.86-1.10)
≥70	1.35 (1.16-1.57)^c^	1.39 (1.22-1.59)^c^
Male	1.17 (1.03-1.34)^c^	1.20 (1.07-1.35)^c^
Race and ethnicity (reference: White)		
Asian or Other Pacific Islander	0.71 (0.60-0.84)^c^	0.76 (0.65-0.88)^c^
Black	1.05 (0.85-1.29)	1.04 (0.86-1.25)
Hispanic or Latinx	0.93 (0.81-1.07)	1.07 (0.94-1.22)
Other^d^	1.19 (0.83-1.69)	1.23 (0.93-1.65)
Disease cause (reference: HCV)		
Chronic HBV	1.36 (1.09-1.69)^c^	1.06 (0.88-1.27)
Nonalcoholic fatty liver disease	1.27 (1.04-1.55)^c^	1.10 (0.91-1.34)
Alcohol-related liver disease	1.52 (1.26-1.84)^c^	1.34 (1.13-1.59)^c^
Other^e^	1.52 (1.30-1.77)	1.46 (1.27-1.68)^c^
Advanced BCLC stage C or D (reference: early BCLC stage 0 or A)	3.28 (2.92-3.69**)**^c^	2.40 (2.15-2.67)^c^
Alpha-fetoprotein, ng/mL (reference: <20)		
20-99	1.40 (1.18-1.67)^c^	1.20 (1.04-1.38**)**^c^
100-999	2.36 (2.02-2.76)^c^	1.72 (1.49-1.97)^c^
≥1000	3.81 (3.29-4.41)^c^	2.84 (2.48-3.25)^c^
Treatment group (reference: curative)^f^		
Noncurative	2.61 (2.04-3.33)^c^	2.51 (2.16-2.90)^c^
No treatment	3.96 (3.06-5.13)^c^	3.15 (2.64-3.76)^c^

Abbreviations: BCLC, Barcelona Clinic Liver Cancer; HBV, hepatitis B virus; HCC, hepatocellular carcinoma; HCV, hepatitis C virus; HR, hazard ratio.

SI conversion factor: To convert alpha-fetoprotein to micrograms per liter, multiply by 1.0.

^a^ N = 3259 unique patients have 1 year of follow-up from the date of HCC diagnosis between 2006 and 2019.

^b^ N = 2168 unique patients have 5 years of follow-up from date of HCC diagnosis between 2006 and 2015.

^c^ *P* <. 05.

^d^ Includes American Indian or Alaska Native, multiracial, and unknown.

^e^ Includes metabolic, autoimmune, cryptogenic, or unknown cause.

^f^ Treatment is modeled as a time-dependent covariate in all models.

### Risk Stratification: Age, Sex, and Race and Ethnicity

Guided by our models, we identified high-risk vs low-risk groups based on clinical and demographic data. Stratifying by AFP level, BCLC stage, and treatment type (eFigure 3A in Supplement 1), we found that patients who had low AFP levels (<20 ng/mL), had early-stage disease (BCLC stage 0 or A) at the time of diagnosis, and who received curative treatment had higher survival rates compared with patients with high AFP levels (≥20 ng/mL), intermediate-to-advanced disease (BCLC stage B, C, or D), and receipt of noncurative or no treatment at 5 years. Stratified by age, sex, and race and ethnicity (eFigure 3B in Supplement 1), we found that patients younger than 70 years of age, female patients, and patients of Asian or Other Pacific Islander race and ethnicity had higher survival rates compared with patients 70 years of age or older, male patients, and patients who were not of Asian or Other Pacific Islander race and ethnicity, respectively.

## Discussion

Current HCC outcomes data are predominantly based on single academic center experiences or large national databases, lacking in scope and granularity. We used comprehensive EHR data from KPNC, one of the largest, most integrated health care systems in the US,^29^ to evaluate survival trends in a large and diverse cohort of patients with HCC. Our findings show that 5-year overall survival for HCC improved significantly over the past decade, particularly for patients with very-early-stage or early-stage disease (BCLC stage 0 or A) receiving curative treatment. We identified several clinical and demographic factors associated with survival, highlighting groups at high and low risk for HCC-related mortality. From a demographic standpoint, patients who are younger (<70 years), female, and of Asian or Other Pacific Islander race and ethnicity represent a more favorable risk group. From a clinical standpoint, patients with low AFP levels, (<20 ng/mL), with early-stage disease (BCLC stage 0 or A), and who received curative treatment had the best overall survival.

With over a decade of patient data and follow-up, our study reveals an overall improvement in HCC survival rates, likely due to earlier detection and better patient selection for a growing armamentarium of treatment options.^30^ This improvement was most pronounced in patients with BCLC stage 0 or A disease and those receiving curative treatments, such as thermal ablation,^31,32,33^ resection,^34,35,36^ and LT.^37,38,39^ The improved survival in era 2 (2013-2019) compared with era 1 (2006-2012) is likely associated, in part, with more patients undergoing curative treatments in the later era. The establishment of adequate future remnant liver criteria for surgical resection in 2012,^40^ with more than 70% 5-year survival,^41^ the increasing use of TARE with Y90 radioembolization to enhance future remnant liver^42^ and enable more surgical resections,^43^ and the increased use of LT as the optimal treatment for patients with unresectable early-stage HCC^41^ were likely associated with this favorable trend.^44^ Moreover, it is well established that aggressive surveillance programs^30^ are crucial for the early detection of HCC and ensuring candidacy for curative treatments,^45,46^ and the relatively high proportion of patients in this cohort with BCLC stage 0 or A disease compared with prior studies^47,48,49^ may be associated with the effectiveness of existing HCC surveillance programs^50,51^ within the KPNC health care system. In addition, improved survival over time was also observed among patients with advanced disease and those receiving no treatment, likely due to advancements in the management of liver disease complications.

The updated BCLC staging system^8^ divides intermediate-stage HCC into 3 treatment subgroups with varying survival estimates. Our study found similar 5-year survival rates for advanced and terminal stages in era 1 and a widening gap in era 2, suggesting that factors beyond diagnosis stage are associated with outcomes. The improvement in survival for patients with intermediate-to-advanced stage HCC in era 2 may be due to adoption of newer treatment paradigms, such as downstaging protocols for LT and newer systemic therapies.

Among patients receiving noncurative treatments, 5-year survival improved from era 1 to era 2. These findings are supported by studies showing that newer treatments, such as TARE, have been associated with higher response rates,^12^ longer time to progression,^12,15^ and improved treatment across HCC stages.^52^ Management of HCC relies on accurate prognostication and careful patient selection for advanced therapies, such as LT. Age younger than 70 years, female sex, and Asian or Other Pacific Islander race and ethnicity were associated with improved survival, with these features indicating the lowest risk for mortality; these criteria may be used for optimizing organs allocated based on estimated outcomes. Older age is associated with worse survival,^20^ including post-LT mortality,^53^ highlighting the importance of considering age in treatment decisions.

In this study, Asian or Other Pacific Islander patients had better outcomes compared with patients who were not Asian or Other Pacific Islander. It is possible that the natural history of HCC in this patient population that largely has HBV-related HCC may be more favorable.^54^ Along with early stage of disease (BCLC stage 0 or A) and receipt of curative treatment, low AFP level (<20 ng/mL) was associated with higher survival in this HCC cohort. Current LT criteria use higher cutoffs, with an AFP level above 1000 ng/mL considered a contraindication to listing^28,55,56^ and improvement in AFP level to less than 500 ng/mL after locoregional therapy as a requirement for subsequent listing at many centers in the US.^57^ However, our study suggests that a lower AFP cutoff of 20 ng/mL is the point at which mortality begins to increase, peaking with an AFP level of higher than 1000 ng/mL carrying the highest mortality risk (AHR, 2.84), which may more accurately inform risk stratification for LT.

### Strengths and Limitations

This study has some strengths. It provides the most comprehensive evaluation of a large cohort of diverse patients with HCC described to date. A major strength is the availability of reliable and granular clinical data accumulated within the same integrated health care system, allowing for a long period of follow-up. Although generalizability to other centers may be limited, KPNC cares for 20% of the population in Northern California and provides a more accurate representation of community-based HCC care.

This study also has some limitations. Given its retrospective nature, the study is subject to bias. Death data were captured using state of California death data for all deaths that occurred prior to 2020, but deaths that occurred during 2020 were captured only if documented within the EHR. Last, given that the study period included patients who received a diagnosis of HCC up to 2019, the effects of newer treatments for HCC, such as TARE, or newer systemic immunotherapies for HCC may be not fully captured.

## Conclusions

Despite modest improvements over the past decade, overall survival among patients with HCC remains low. The trends observed in this large, racially and ethnically diverse patient cohort clearly demonstrate the benefits of early detection, as patients with early-stage disease who received curative treatments had the best survival; this effect became more pronounced in recent years. This study also highlights important demographic factors associated with favorable survival, including age younger than 70 years, female sex, and Asian or Other Pacific Islander race and ethnicity, which, taken together, may inform treatment allocation, particularly with respect to LT.

## Notes

Supplement 1. **eTable 1.** Univariate Cox Regression Analysis of Race and Ethnicity, Socioeconomic Status, and Disease Factors Associated With All-Cause Mortality Among Patients With 1- and 5-Year of Follow-Up From HCC Diagnosis **eTable 2.** Univariate Cox Regression Analysis of Race/Ethnicity, Socioeconomic Status, and Disease Factors Associated With HCC-Specific Mortality Among Patients With 1- and 5-Year of Follow-up From HCC Diagnosis **eTable 3.** Multivariable Cox Proportional Hazard Regression of HCC-Specific Mortality with 1- and 5-Years of Follow-up From HCC Diagnosis **eFigure 1A.** Kaplan-Meier Survival Curves for BCLC 0/A Stage Stratified by Diagnosis Era **eFigure 1B.** Kaplan-Meier Survival Curves for BCLC B Stage Stratified by Diagnosis Era **eFigure 1C.** Kaplan-Meier Survival Curves for BCLC C Stage Stratified by Diagnosis Era **eFigure 1D.** Kaplan-Meier Survival Curves for BCLC D Stage Stratified by Diagnosis Era **eFigure 2A.** Kaplan-Meier Survival Curve of Curative Treatment Group by Era **eFigure 2B.** Kaplan-Meier Survival Curve of Non-Curative Treatment Group by Era **eFigure 2C.** Kaplan-Meier Survival Curve of No Treatment Group by Era **eFigure 3A.** Risk Stratification Based on AFP, BCLC Stage, and Treatment **eFigure 3B.** Risk Stratification Based on Age, Sex, and Race/Ethnicity

Supplement 2.
Data Sharing Statement
