# Supplementary material for: Survival Outcomes Among Patients With Hepatocellular Carcinoma in a Large Integrated US Health System
Source: JAMA Netw Open. 2024 Sep 24;7(9):e2435066. doi: 10.1001/jamanetworkopen.2024.35066 (PMC11423175; doi:10.1001/jamanetworkopen.2024.35066)
Supplement: Supplement 1. — eTable 1. Univariate Cox Regression Analysis of Race and Ethnicity, Socioeconomic Status, and Disease Factors Associated With All-Cause Mortality Among Patients With 1- and 5-Year of Follow-Up From HCC Diagnosis eTable 2. Univariate Cox Regression Analysis of Race/Ethnicity, Socioeconomic Status, and Disease Factors Associated With HCC-Specific Mortality Among Patients With 1- and 5-Year of Follow-up From HCC Diagnosis eTable 3. Multivariable Cox Proportional Hazard Regression of HCC-Specific Mortality with 1- and 5-Years of Follow-up From HCC Diagnosis eFigure 1A. Kaplan-Meier Survival Curves for BCLC 0/A Stage Stratified by Diagnosis Era eFigure 1B. Kaplan-Meier Survival Curves for BCLC B Stage Stratified by Diagnosis Era eFigure 1C. Kaplan-Meier Survival Curves for BCLC C Stage Stratified by Diagnosis Era eFigure 1D. Kaplan-Meier Survival Curves for BCLC D Stage Stratified by Diagnosis Era eFigure 2A. Kaplan-Meier Survival Curve of Curative Treatment Group by Era eFigure 2B. Kaplan-Meier Survival Curve of Non-Curative Treatment Group by Era eFigure 2C. Kaplan-Meier Survival Curve of No Treatment Group by Era eFigure 3A. Risk Stratification Based on AFP, BCLC Stage, and Treatment eFigure 3B. Risk Stratification Based on Age, Sex, and Race/Ethnicity [file jamanetwopen-e2435066-s001.pdf]

Supplementary Online Content

Yilma M, Houhong Xu R, Saxena V, et al. Survival outcomes among patients with hepatocellular carcinoma in a large integrated US health system. *JAMA Netw Open.* 2024;7(9):e2435066. doi:10.1001/jamanetworkopen.2024.35066

eTable 1. Univariate Cox Regression Analysis of Race and Ethnicity, Socioeconomic Status, and Disease Factors Associated With All-Cause Mortality Among Patients With 1- and 5-Year of Follow-Up From HCC Diagnosis
eTable 2. Univariate Cox Regression Analysis of Race/Ethnicity, Socioeconomic Status, and Disease Factors Associated With HCC-Specific Mortality Among Patients With 1- and 5-Year of Follow-up From HCC Diagnosis
eTable 3. Multivariable Cox Proportional Hazard Regression of HCC-Specific Mortality with 1- and 5-Years of Follow-up From HCC Diagnosis
eFigure 1A. Kaplan-Meier Survival Curves for BCLC 0/A Stage Stratified by Diagnosis Era
eFigure 1B. Kaplan-Meier Survival Curves for BCLC B Stage Stratified by Diagnosis Era
eFigure 1C. Kaplan-Meier Survival Curves for BCLC C Stage Stratified by Diagnosis Era
eFigure 1D. Kaplan-Meier Survival Curves for BCLC D Stage Stratified by Diagnosis Era
eFigure 2A. Kaplan-Meier Survival Curve of Curative Treatment Group by Era
eFigure 2B. Kaplan-Meier Survival Curve of Non-Curative Treatment Group by Era
eFigure 2C. Kaplan-Meier Survival Curve of No Treatment Group by Era
eFigure 3A. Risk Stratification Based on AFP, BCLC Stage, and Treatment
eFigure 3B. Risk Stratification Based on Age, Sex, and Race/Ethnicity

This supplementary material has been provided by the authors to give readers additional information about their work.

© 2024 Yilma M et al. *JAMA Network Open.*

eTable 1. Univariate Cox Regression Analysis of Race and Ethnicity, Socioeconomic Status, and Disease Factors Associated With All-Cause Mortality Among Patients With 1- and 5-Year of Follow-Up From HCC Diagnosis

	Unadjusted HR (95% CI)	
**Variables**	**1-year follow-up**[a]	**5-years follow-up**[b]
**Age at HCC Diagnosis** (*ref: 40-<60 years)*		
18-<40 years	0.98 (0.55-1.61)	0.90 (0.53-1.43)
60-<70 years	0.89 (0.78-1.02)	1.01 (0.89-1.14)
≥70 years	1.49 (1.30-1.69)[f]	1.61 (1.43-1.82)[f]
**Male**	1.04 (0.92-1.18)	1.08 (0.96-1.22)
**Race and Ethnicity** (*ref = white patients*)		
Asian/Pacific Islander patients	0.73 (0.64-0.84)[f]	0.76 (0.67-0.87)[f]
Black patients	1.03 (0.85-1.24)	1.03 (0.86-1.23)
Hispanic/Latino patients	0.94 (0.81-1.07)	0.98 (0.86-1.12)
Other[c]	0.92 (0.64-1.27)	1.02 (0.96-1.22)
**Disease Etiology** (*ref = HCV*)		
Chronic HBV	0.99 (0.83-1.17)	0.81 (0.69-0.95)[f]
NAFLD	1.25 (1.02-1.51)[f]	1.21 (1.00-1.46)
ALD	1.58 (1.30-1.89)[f]	1.47 (1.23-1.74)[f]
Other[d]	2.17 (1.90-2.48)[f]	1.88 (1.66-2.13)[f]
**FIB-4>3.25 or Cirrhosis Diagnosis**	1.43 (1.26-1.64)[f]	1.36 (1.21-1.54)[f]
**Advanced BCLC** (*ref = early*)	4.46 (4.00-4.97)[f]	3.24 (2.91-3.61)[f]
**Alpha-fetoprotein (AFP)** (*ref = <20 ng/mL*)		
20 - 99 ng/mL	1.31 (1.09-1.56)[f]	1.18 (1.01-1.36)[f]
100 - 999 ng/mL	2.60 (2.22-3.04)[f]	1.89 (1.63-2.17)[f]
≥ 1000	5.11 (4.43-5.90)[f]	3.35 (2.91-3.85)[f]
**Treatment Group** (*ref = curative*)[e]		
Non-curative	4.15 (3.27-5.26)[f]	3.27 (2.83-3.76)[f]
No Treatment	6.17 (4.81-7.90)[f]	4.36 (3.69-5.15)[f]

[a]N = 3259 unique patients have 1-years of follow-up from the date of HCC diagnosis.
[b]N = 2168 unique patients have 5-years of follow-up from date of HCC diagnosis.
[c]"Other" race/ethnicity includes multiracial, Native American/Alaskan native, and unknown race/ethnicity.
[d]"Other" etiology includes metabolic, autoimmune, cryptogenic, unknown etiology.
[e]Treatment is modelled as a time-dependent covariate in all models.
[f]p-value < 0.05.

© 2024 Yilma M et al. *JAMA Network Open.*

eTable 2. Univariate Cox Regression Analysis of Race/Ethnicity, Socioeconomic Status, and Disease Factors Associated With HCC-Specific Mortality Among Patients With 1- and 5-Year of Follow-up From HCC Diagnosis

Variables	Unadjusted HR (95% CI)	
	1-year follow-up[a]	5-years follow-up[b]
**Age at HCC Diagnosis** (*ref: 40-<60 years)*		
18-<40 years	1.18 (0.64-1.97)	1.14 (0.65-1.84)
60-<70 years	0.87 (0.74-1.02)	1.01 (0.88-1.17)
≥70 years	1.62 (1.40-1.88)[f]	1.76 (1.53-2.01)[f]
**Male**	1.11 (0.96-1.28)	1.14 (0.99-1.30)
**Race and Ethnicity** (*ref = white patients*)		
Asian/Pacific Islander patients	0.77 (0.66-0.90)[f]	0.79 (0.69-0.92)[f]
Black patients	1.08 (0.87-1.32)	1.07 (0.87-1.30)
Hispanic/Latino patients	0.90 (0.77-1.05)	0.95 (0.82-1.11)
Other[c]	0.97 (0.65-1.38)	0.81 (0.55-1.15)
**Disease Etiology** (*ref = HCV*)		
Chronic HBV	1.12 (0.92-1.35)	0.93 (0.78-1.11)
NAFLD	1.35 (1.09-1.67)[f]	1.30 (1.04-1.61)[f]
ALD	1.52 (1.23-1.88)[f]	1.41 (1.14-1.72)[f]
Other[d]	2.40 (2.07-2.78)[f]	2.07 (1.80-2.39)[f]
**FIB-4>3.25 or Cirrhosis Diagnosis**	1.28 (1.11-1.48)[f]	1.25 (1.10-1.43)[f]
**Advanced BCLC** (*ref = early*)	4.67 (4.13-5.27)[f]	3.41 (3.02-3.85)[f]
**Alpha-fetoprotein (AFP)** (*ref = <20 ng/mL*)		
20 - 99 ng/mL	1.41 (1.15-1.74)[f]	1.22 (1.02-1.45)[f]
100 - 999 ng/mL	3.07 (2.57-3.67)[f]	2.17 (1.84-2.54)[f]
≥ 1000	6.18 (5.26-7.29)[f]	3.95 (3.38-4.62)[f]
**Treatment Group** (*ref = curative*)[e]		
Non-curative	5.22 (3.90-6.98)[f]	3.73 (3.16-4.42)[f]
No Treatment	7.38 (5.46-9.99)[f]	4.62 (3.79-5.62)[f]

[a]N = 3259 unique patients have 1-years of follow-up from the date of HCC diagnosis.
[b]N = 2168 unique patients have 5-years of follow-up from date of HCC diagnosis.
[c]"Other" race/ethnicity includes multiracial, Native American/Alaskan native, and unknown race/ethnicity.
[d]"Other" etiology includes metabolic, autoimmune, cryptogenic, unknown etiology.
[e]Treatment is modelled as a time-dependent covariate in all models.
[f]p-value < 0.05

© 2024 Yilma M et al. *JAMA Network Open.*

eTable 3. Multivariable Cox Proportional Hazard Regression of HCC-Specific Mortality with 1- and 5-Years of Follow-up From HCC Diagnosis

Variables	Adjusted HR (95% CI)	
	1-year follow-up[a]	5-years follow-up[b]
**Age at HCC Diagnosis** (*ref: 40-<60 years)*		
18-<40 years	1.11 (0.60-2.05)	0.99 (0.57-1.70)
60-<70 years	0.91 (0.77-1.07)	0.96 (0.83-1.10)
≥70 years	1.38 (1.17-1.63)[f]	1.46 (1.26-1.70)[f]
**Male**	1.29 (1.11-1.50)[f]	1.25 (1.09-1.44)[f]
**Race and Ethnicity** (*ref = white patients*)		
Asian/Pacific Islander patients	0.70 (0.57-0.84)[f]	0.75 (0.63-0.89)[f]
Black patients	1.10 (0.87-1.38)	1.04 (0.84-1.29)
Hispanic/Latino patients	0.90 (0.76-1.06)	1.05 (0.90-1.21)
Other[3]	1.28 (0.87-1.89)	1.04 (0.72-1.50)
**Disease Etiology** (*ref = HCV*)		
Chronic HBV	1.54 (1.21-1.96)[f]	1.20 (0.97-1.48)
NAFLD	1.41 (1.13-1.78)[f]	1.16 (0.94-1.45)
ALD	1.56 (1.25-1.94)[f]	1.19 (0.97-1.46)
Other[4]	1.66 (1.39-1.97)[f]	1.53 (1.30-1.79)[f]
**Advanced BCLC** (*ref = early*)	3.30 (2.90-3.76)[f]	2.49 (2.20-2.81)[f]
**Alpha-fetoprotein (AFP)** (*ref = <20 ng/mL*)		
20 – 99 ng/mL	1.52 (1.24-1.87)[f]	1.27 (1.08-1.51)[f]
100 – 999 ng/mL	2.82 (2.36-3.38)[f]	1.99 (1.70-2.33)[f]
≥ 1000 ng/mL	4.62 (3.90-5.46)[f]	3.25 (2.79-3.80)[f]
**Treatment Group** (*ref = curative*)[5]		
Non-curative	3.19 (2.36-4.31)[f]	2.78 (2.34-3.32)[f]
No Treatment	4.66 (3.40-6.39)[f]	3.26 (2.65-4.01)[f]

[a]N = 3259 unique patients have 1-years of follow-up from the date of HCC diagnosis between 2006 and 2019.

[b]N = 2168 unique patients have 5-years of follow-up from date of HCC diagnosis between 2006 and 2015.

[c]"Other" race/ethnicity includes multiracial, Native American/Alaskan native, and unknown race/ethnicity.

[d]"Other" etiology includes metabolic, autoimmune, cryptogenic, unknown etiology.

[e]Treatment is modelled as a time-dependent covariate in all models.

[f]p-value < 0.05

© 2024 Yilma M et al. *JAMA Network Open.*

eFigure 1A. Kaplan-Meier Survival Curves for BCLC 0/A Stage Stratified by Diagnosis Era

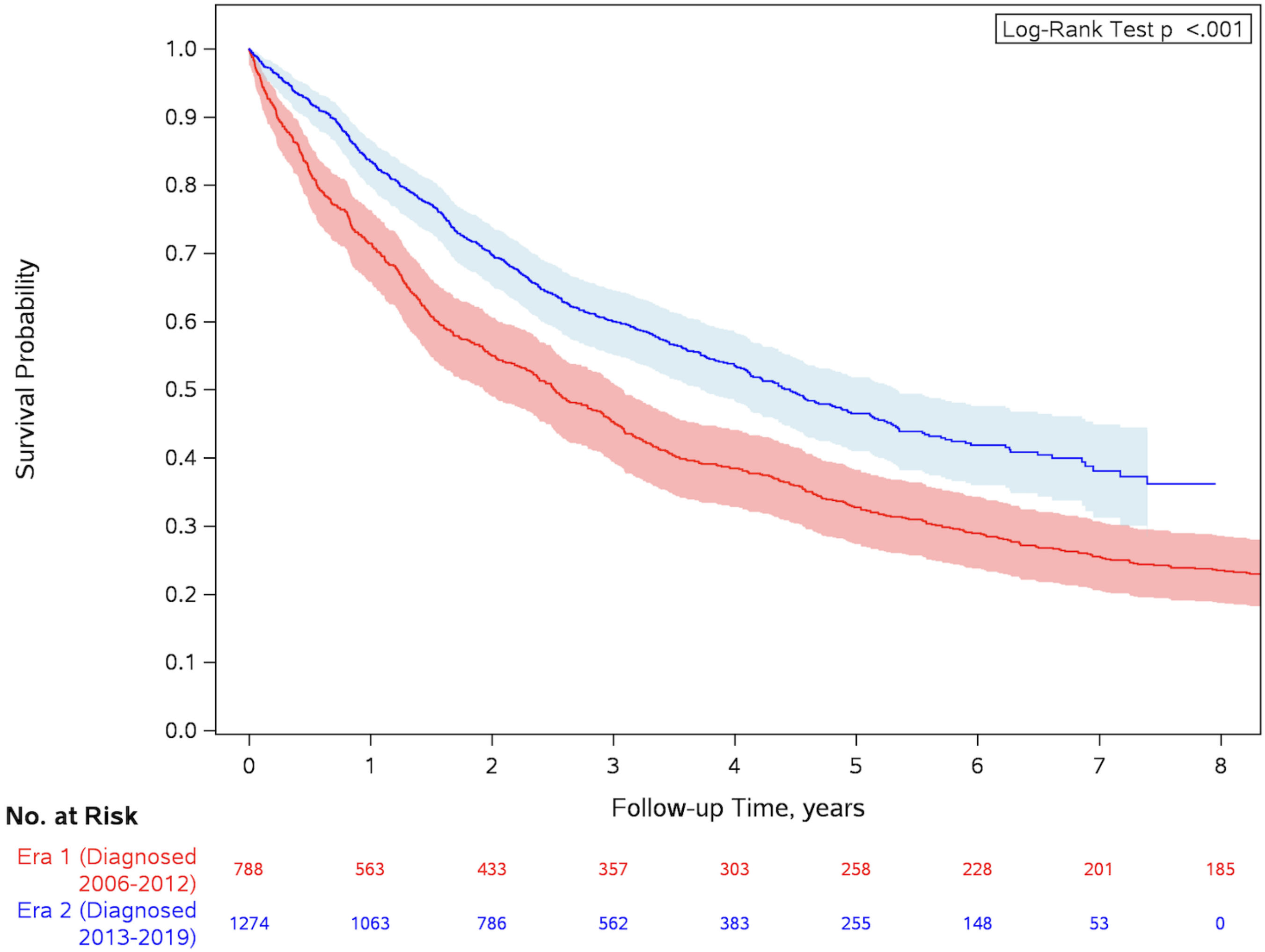

Plots are truncated after 8 years of follow-up. Median survival and 95% confidence interval are calculated based on all patients who are alive with minimal of follow-up of 1 year and maximal follow-up of 15 years.

© 2024 Yilma M et al. *JAMA Network Open.*

eFigure 1B. Kaplan-Meier Survival Curves for BCLC B Stage Stratified by Diagnosis Era

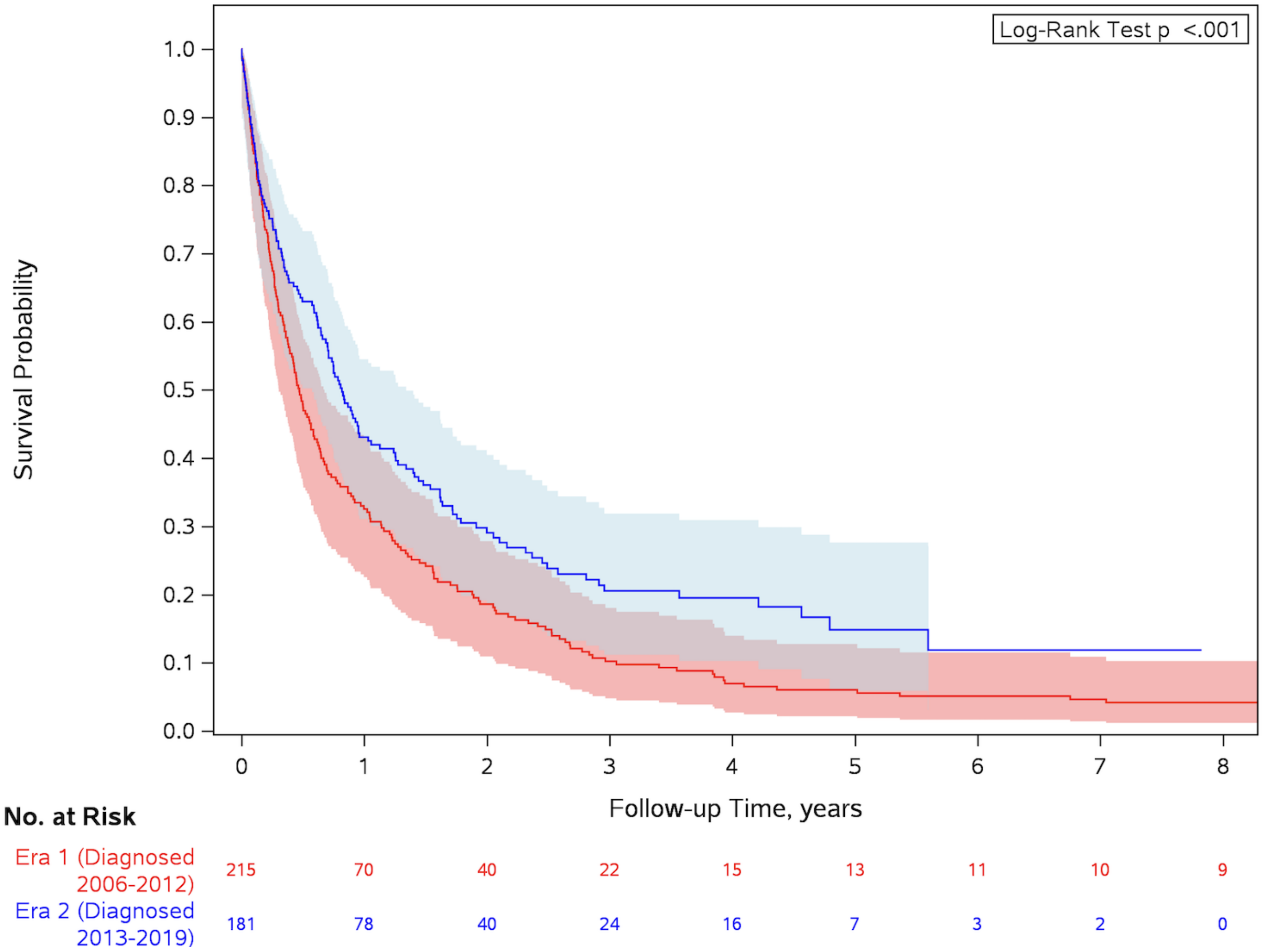

Plots are truncated after 8 years of follow-up. Median survival and 95% confidence interval are calculated based on all patients who are alive with minimal of follow-up of 1 year and maximal follow-up of 15 years.

© 2024 Yilma M et al. *JAMA Network Open.*

eFigure 1C. Kaplan-Meier Survival Curves for BCLC C Stage Stratified by Diagnosis Era

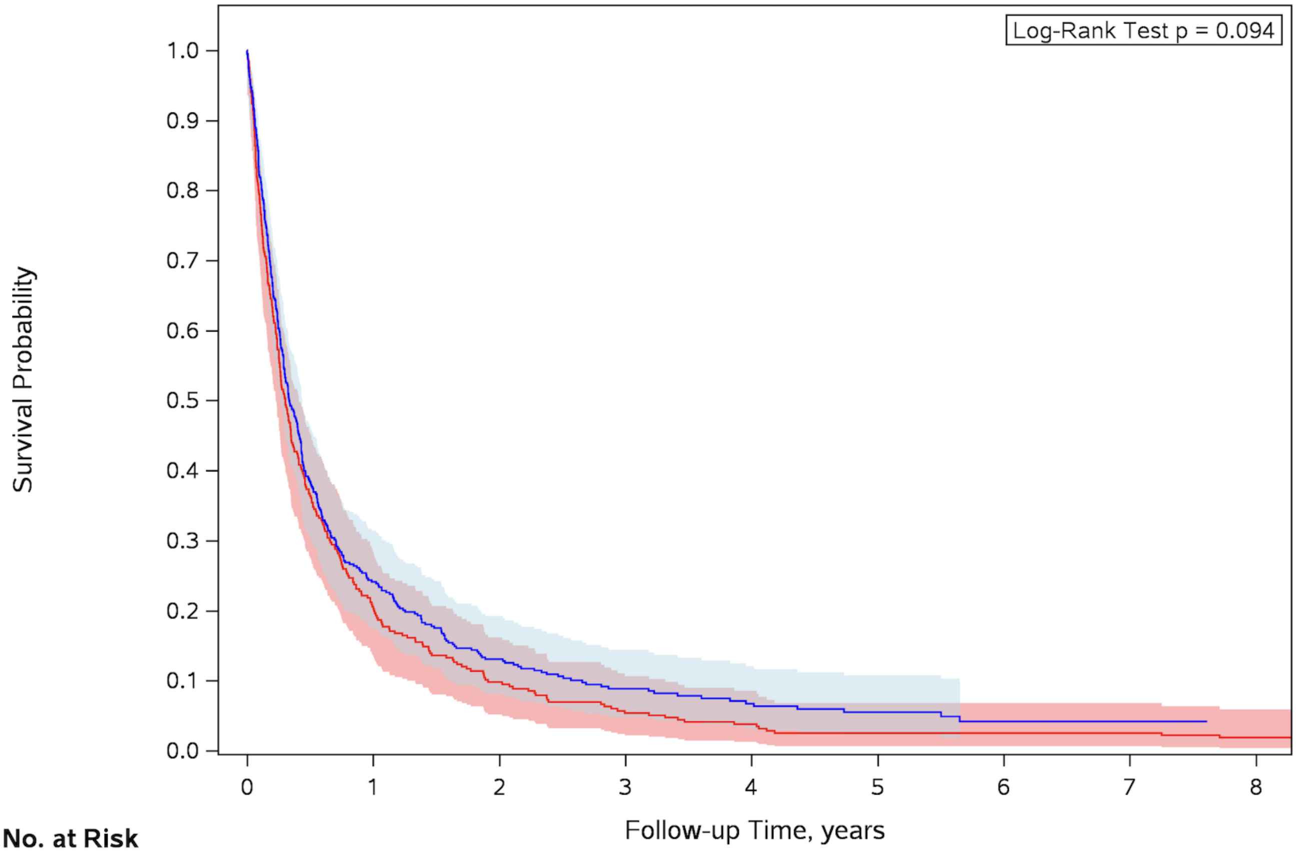

No. at Risk	0	1	2	3	4	5	6	7	8
Era 1 (Diagnosed 2006-2012)	316	65	31	17	12	8	8	8	6
Era 2 (Diagnosed 2013-2019)	398	96	49	30	18	11	5	1	0

Plots are truncated after 8 years of follow-up. Median survival and 95% confidence interval are calculated based on all patients who are alive with minimal of follow-up of 1 year and maximal follow-up of 15 years.

© 2024 Yilma M et al. *JAMA Network Open.*

eFigure 1D. Kaplan-Meier Survival Curves for BCLC D Stage Stratified by Diagnosis Era

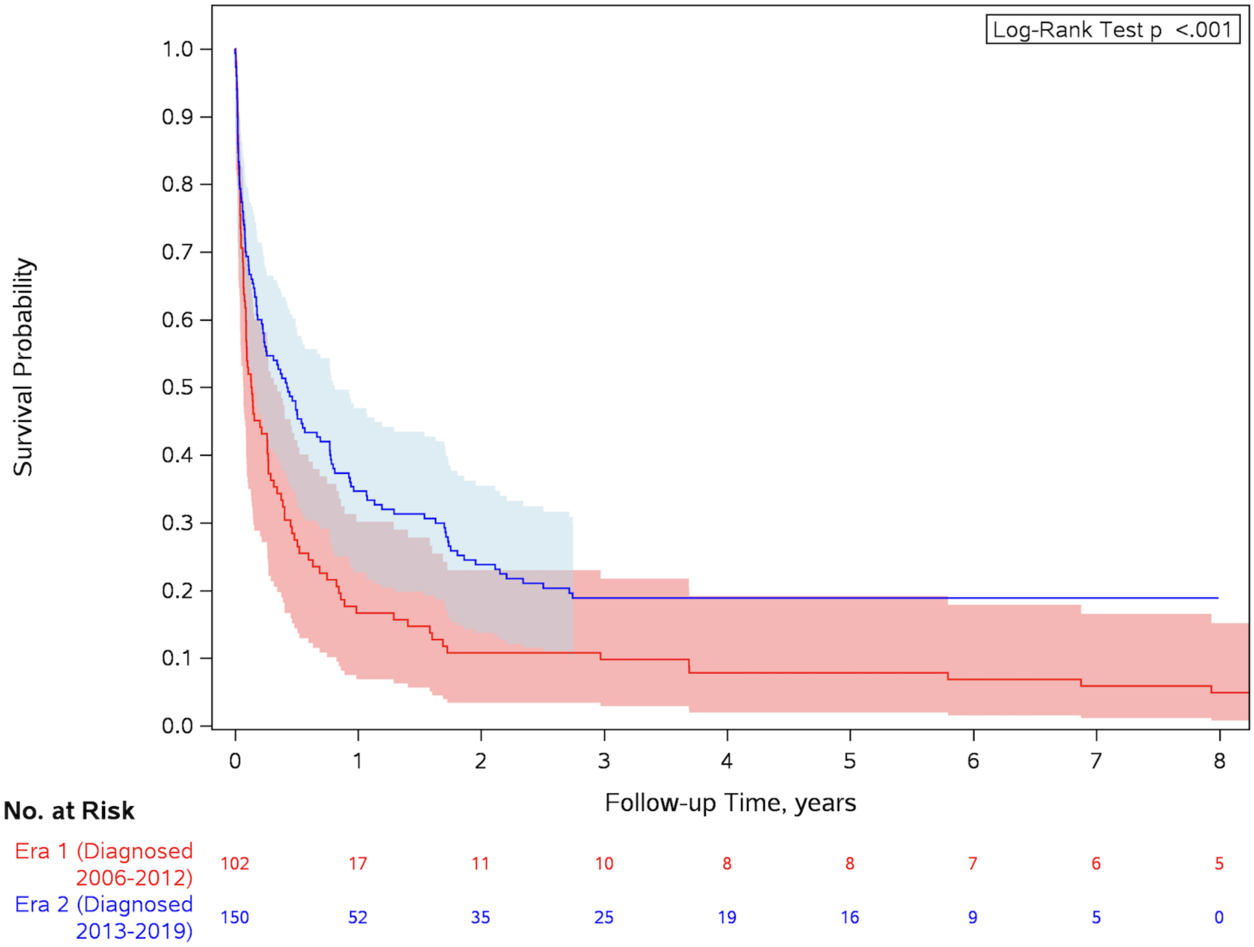

Plots are truncated after 8 years of follow-up. Median survival and 95% confidence interval are calculated based on all patients who are alive with minimal of follow-up of 1 year and maximal follow-up of 15 years.

© 2024 Yilma M et al. *JAMA Network Open.*

eFigure 2A. Kaplan-Meier Survival Curve of Curative Treatment Group by Era

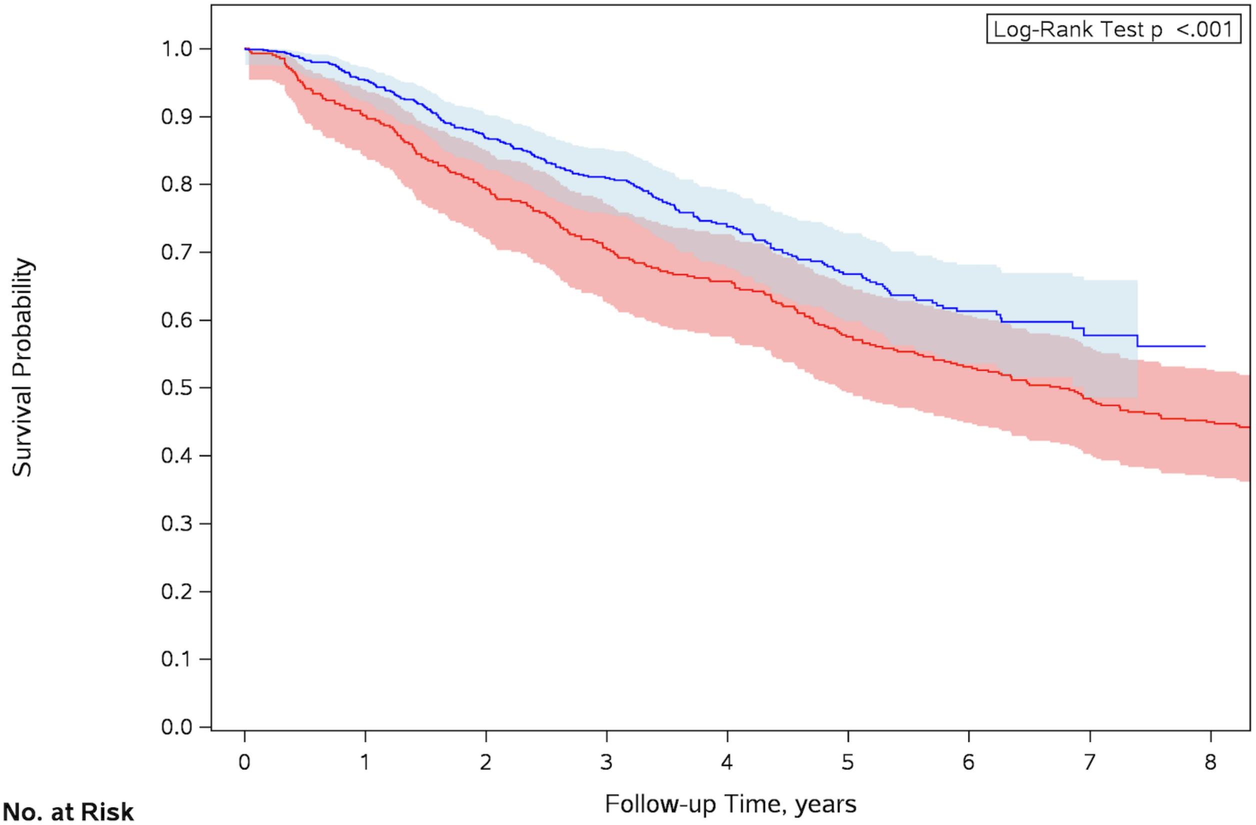

No. at Risk

	0	1	2	3	4	5	6	7	8
Era 1 (Diagnosed 2006-2012)	405	365	321	286	266	233	215	196	182
Era 2 (Diagnosed 2013-2019)	790	753	618	490	338	235	135	53	0

Plots are truncated after 8 years of follow-up. Median survival and 95% confidence interval are calculated based on all patients who are alive with minimal of follow-up of 1 year and maximal follow-up of 15 years.

© 2024 Yilma M et al. *JAMA Network Open.*

eFigure 2B. Kaplan-Meier Survival Curve of Non-Curative Treatment Group by Era

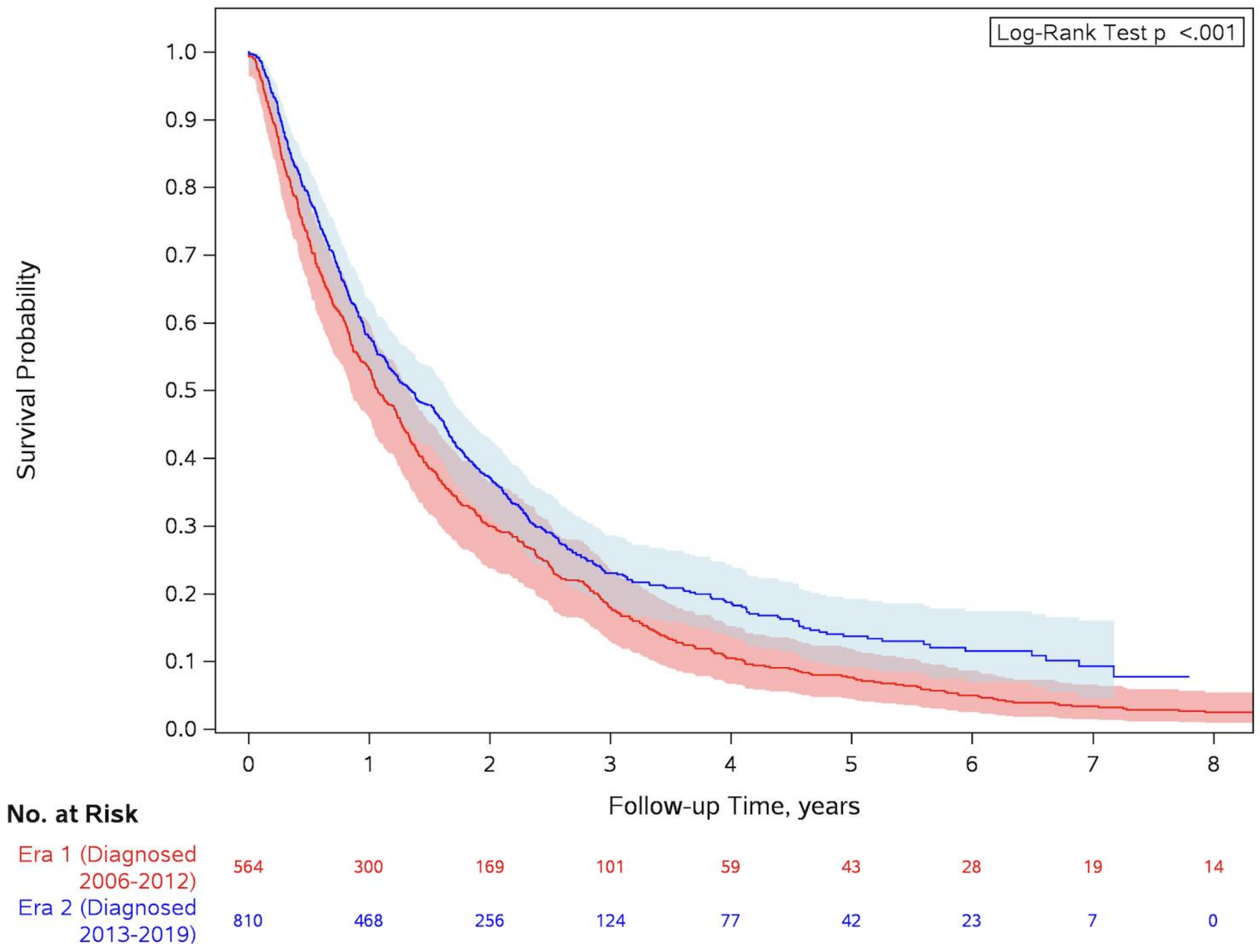

Plots are truncated after 8 years of follow-up. Median survival and 95% confidence interval are calculated based on all patients who are alive with minimal of follow-up of 1 year and maximal follow-up of 15 years.

© 2024 Yilma M et al. *JAMA Network Open.*

eFigure 2C. Kaplan-Meier Survival Curve of No Treatment Group by Era

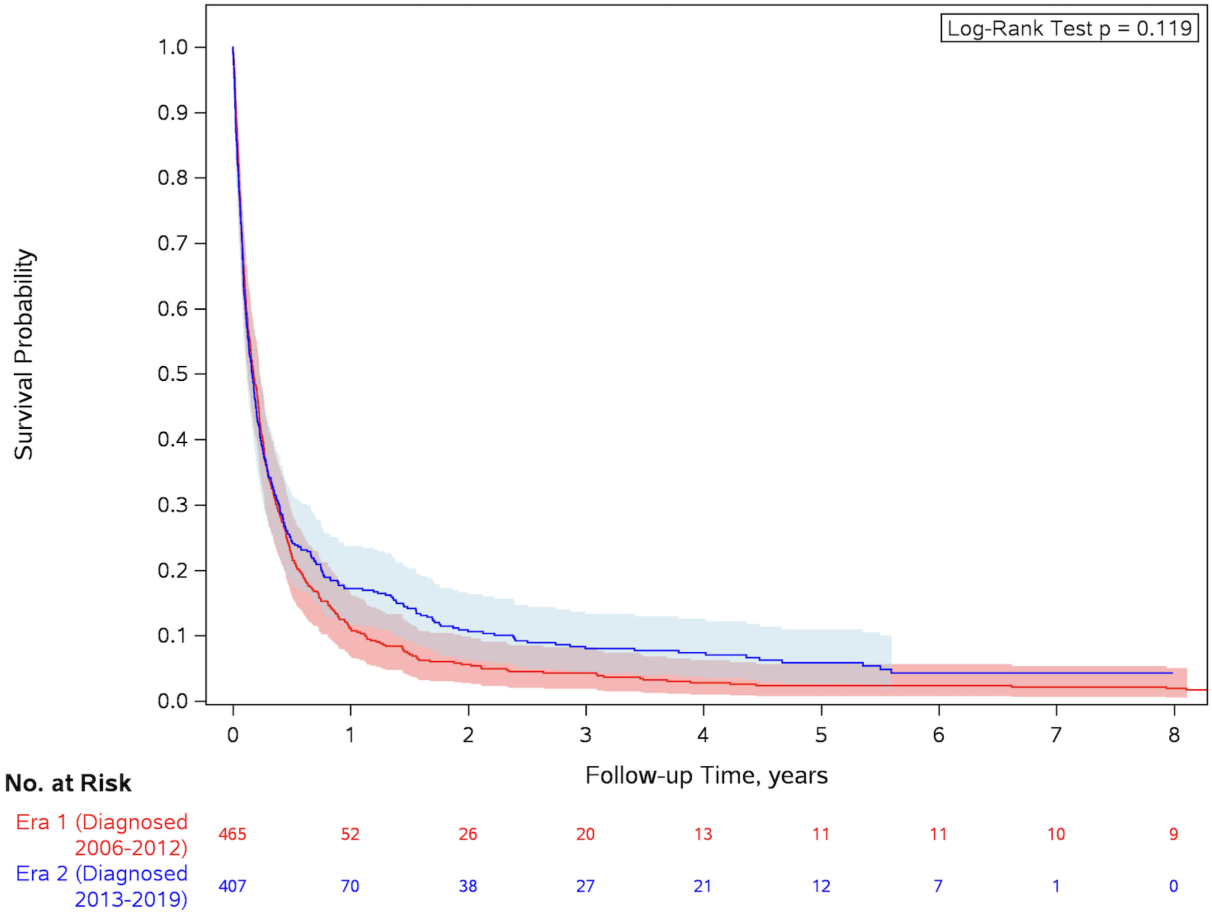

Plots are truncated after 8 years of follow-up. Median survival and 95% confidence interval are calculated based on all patients who are alive with minimal of follow-up of 1 year and maximal follow-up of 15 years.

© 2024 Yilma M et al. *JAMA Network Open.*

eFigure 3A. Risk Stratification Based on AFP, BCLC Stage, and Treatment

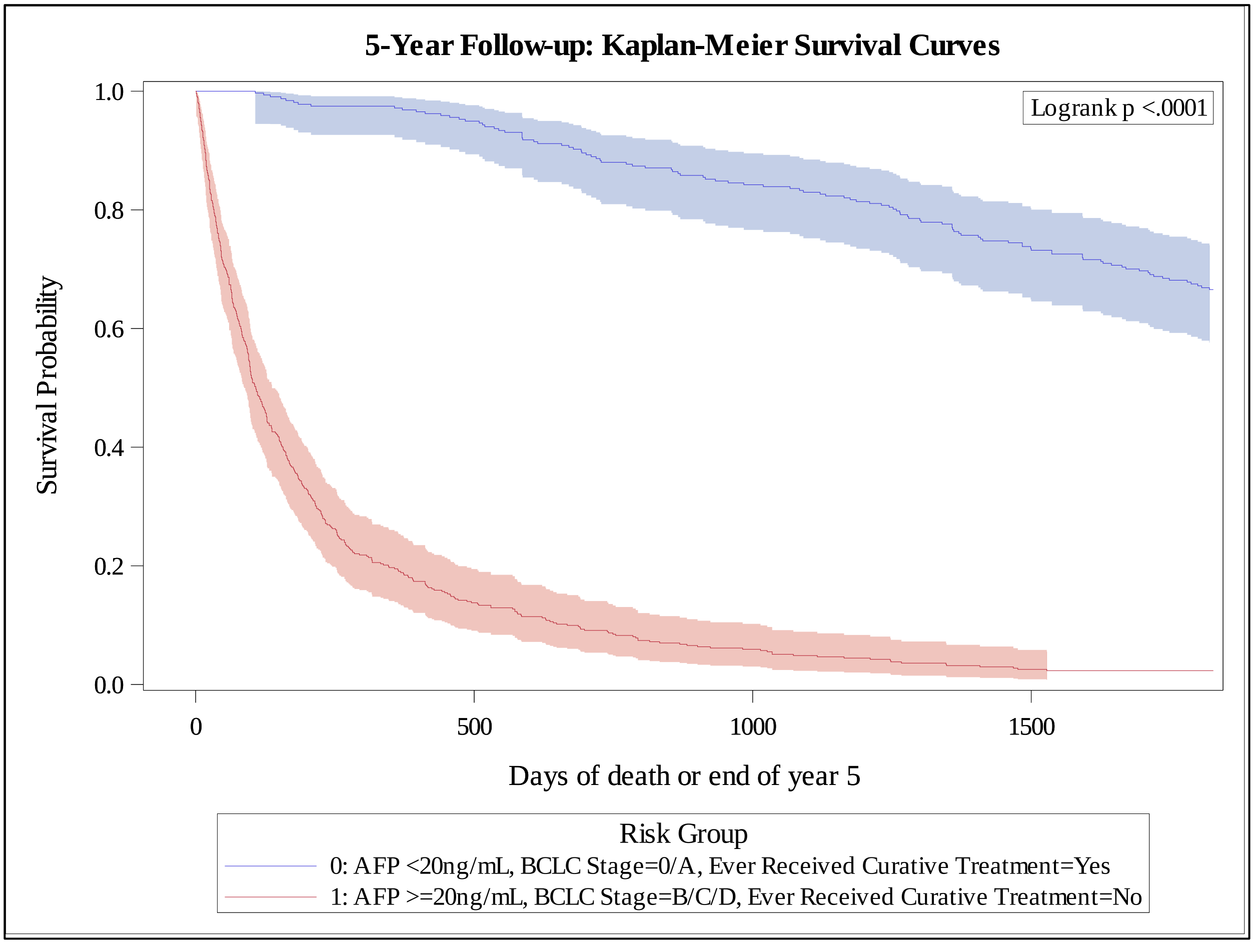

© 2024 Yilma M et al. *JAMA Network Open.*

eFigure 3B. Risk Stratification Based on Age, Sex, and Race/Ethnicity

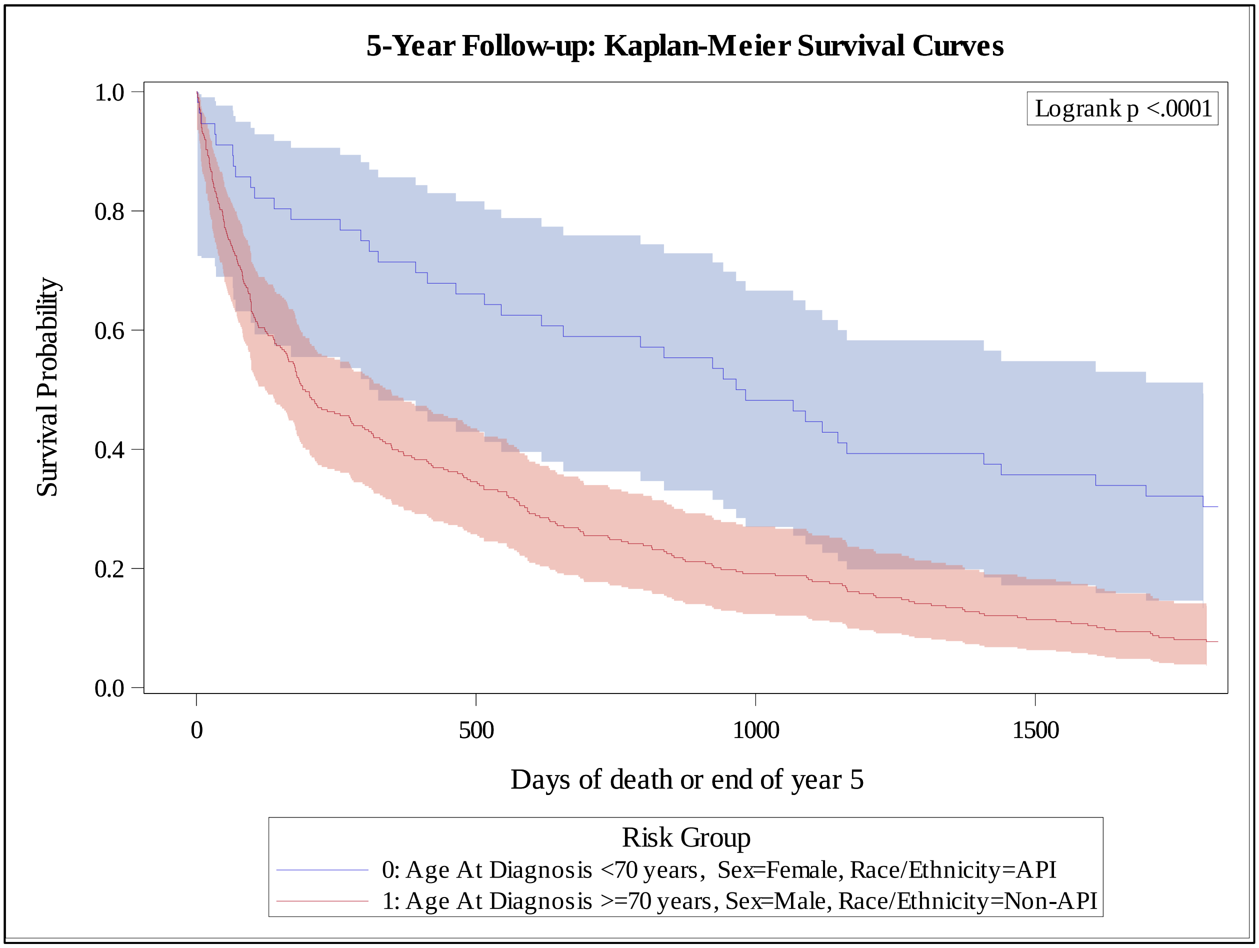

© 2024 Yilma M et al. *JAMA Network Open.*
