# Supplementary material for: Survival Outcomes Among Patients With Hepatocellular Carcinoma in a Large Integrated US Health System
Source: JAMA Netw Open. 2024 Sep 24;7(9):e2435066. doi: 10.1001/jamanetworkopen.2024.35066 (PMC11423175; doi:10.1001/jamanetworkopen.2024.35066)
Supplement: Supplement 2. — Data Sharing Statement [file jamanetwopen-e2435066-s002.pdf]

Data Sharing Statement

Yilma. Survival Outcomes Among Patients With Hepatocellular Carcinoma in a Large Integrated US Health System. *JAMA Netw Open*. Published September 24, 2024. doi:10.1001/jamanetworkopen.2024.35066

Data
Data available: No
